# TELEMAC modelling of the influence of the Poyang Lake Hydraulic Project on the habitat of *Vallisneria natans*

**DOI:** 10.1038/s41598-022-11314-5

**Published:** 2022-05-04

**Authors:** Yang Xiao, Zixuan Wang, Taotao Zhang, Dongfang Liang, Ran Gu, Kang Yuan

**Affiliations:** 1 State Key Laboratory of Hydrology, Water Resources and Hydraulic Engineering, Hohai University, Nanjing, China; 2 College of Water Conservancy and Hydropower Engineering, Hohai University, Nanjing, China; 3 Institute of Water Science and Technology, Hohai University, Nanjing, China; 4 Yangtze Institute for Conservation and Development, Hohai University, Nanjing, China; 5 Department of Engineering, University of Cambridge, Cambridge, CB2 1PZ UK

**Keywords:** Ecology, Environmental sciences, Hydrology, Limnology

## Abstract

The Poyang Lake Hydraulic Project (PLHP) has been proposed to address the water resource shortage and hydro-environment deterioration in Poyang Lake. This proposal has raised concerns over the possible changes to the habitat of aquatic organisms. *Vallisneria natan*s is a main food source for the Siberian Crane, an indicator species for migratory birds in Poyang Lake. In this study, the influence of the PLHP on the habitat suitability of *Vallisneria natans* is predicted based on a hydrodynamic model and the growth characteristics of *Vallisneria natans*. The results show that the effect of the PLHP varies greatly in different typical years. The mean monthly habitat area of *Vallisneria natans* can increase by up to 191% in a low-water-level year, 145% in a medium-water-level year, yet only 18% in a high-water-level year. The habitat area can reach more than 1000 km^2^ during most of September and October, nearly 1/3 of the total area of the lake region. It indicates that *Vallisneria natans* will gain large areas of land suitable for its growth, and provide abundant food sources for Siberian Crane during winter. These findings can be helpful to evaluate the ecological benefits of the regulatory schemes of the PLHP.

## Introduction

Lakes are crucial not only for water systems, but also for maintaining biodiversity and ecological balance. In order to ensure the social and ecological benefits of the lakes^1,2^, various hydraulic projects have been constructed to regulate flow, conserve water, and ensure navigation and water supply worldwide^3^. Nevertheless, the operation of these projects will inevitably impact the hydrodynamic conditions of the lakes, resulting in complicated alterations to the lake floodplains, which are important habitats for wetland biotas^4^.

Poyang Lake is the largest freshwater lake in China and the largest wintering habitat for birds in Asia. It hosts as many as 155 species of migratory birds, many of which are rare species. Recently, the middle and lower reaches of the Yangtze River have experienced rapid urbanization, reservoir construction and climate change^5^. Consequently, the river forcing on Poyang Lake weakened, allowing more lake water to flow into the river^6,7^, causing the dry-season water level to drop and the dry-season period to extend in Poyang Lake^8,9^. To alleviate these adverse trends, the Jiangxi Province Government proposed the construction of the Poyang Lake Hydraulic Project (PLHP) on the waterway connecting the Poyang Lake and the Yangtze River. The operation of the PLHP will inevitably influence the hydrodynamic conditions in the surrounding area, which is sensitive and controversial^10^. Many scientists believe that the increased water storage will reduce the flow speed in the lake and increase the eutrophication risk. Furthermore, the PLHP could have a negative impact on some wintering grounds for migratory birds, which may drive some rare species into extinction, such as Siberian Crane. While supporters claim that such concerns are overstated, the negative impact on ecology should be avoided through scientific regulations. Therefore, it is necessary to systematically evaluate and optimize the operation of the PLHP.

In recent years, some potential influences of the PLHP on different aspects of hydro-environment have been reported. Using the Environmental Fluid Dynamics Code (EFDC) model, Wang et al.^3^ and Lai et al.^11^ predicted that the flow velocity in the northern part of the lake would reduce by about 50% when doubling the water storage. Ho et al.^12^ established a 1D and 3D coupled numerical model to simulate the hydro-environmental evolutions for different operations of the PLHP and found that the pollutant and dissolved oxygen concentration would be reduced as the water level rose during the dry season. Such changes could threaten the survival of aquatic wildlife and endanger their living habitats. Han et al.^13^ reported that the PLHP would increase the living area and food resources for the local fish, while opposite would be the case for the migratory fish. Some studies have also been conducted on the changes to the habitat for migratory birds in Poyang Lake after the operation of the PLHP. Through numerical simulation, Yao et al.^14^ and Zhu et al.^15^ revealed that there would be an increased area suitable for migratory birds and the ecological stress could be alleviated by optimizing the PLHP operation during dry years. In previous studies^16–18^, Siberian Crane was commonly regarded as the indicator species among the wintering migratory birds in Poyang Lake. According to relevant research^19^, there are few alternative habitats for these birds, and nearly 98% of Siberian Crane migrate to the Poyang Lake every winter. They often arrive in November and depart in the following March, feeding mainly on the tubers of *Vallisneria natans*^20^. It has been reported that when tubers collapse, Siberian Crane will be forced to change their foods and feeding areas^21^. These shifts will create competition among species whose original feeding habits do not overlap. Considering the critically endangered status of Siberian Crane in International Union for the Conservation of Nature and Natural Resources (IUCN), any additional stress would be considered negative and detrimental. Therefore, the abundance of *Vallisneria natans* is crucial for protecting the living conditions of this bird. However, there have been few studies about the influence of the PLHP on *Vallisneria natans*.

In this research, a two-dimensional hydrodynamic model of Poyang Lake was established using TELEMAC-2D software, taking into account the situation with and without the PLHP. The habitat suitability of *Vallisneria natans* before and after the operation of the PLHP were investigated, with quantitative analyses of the changes in the habitat suitability. The findings can provide technical support for evaluating the benefits of the PLHP and designing its operation schemes.

## Overview of Poyang Lake and the PLHP

### Study area

**Figure 1:**
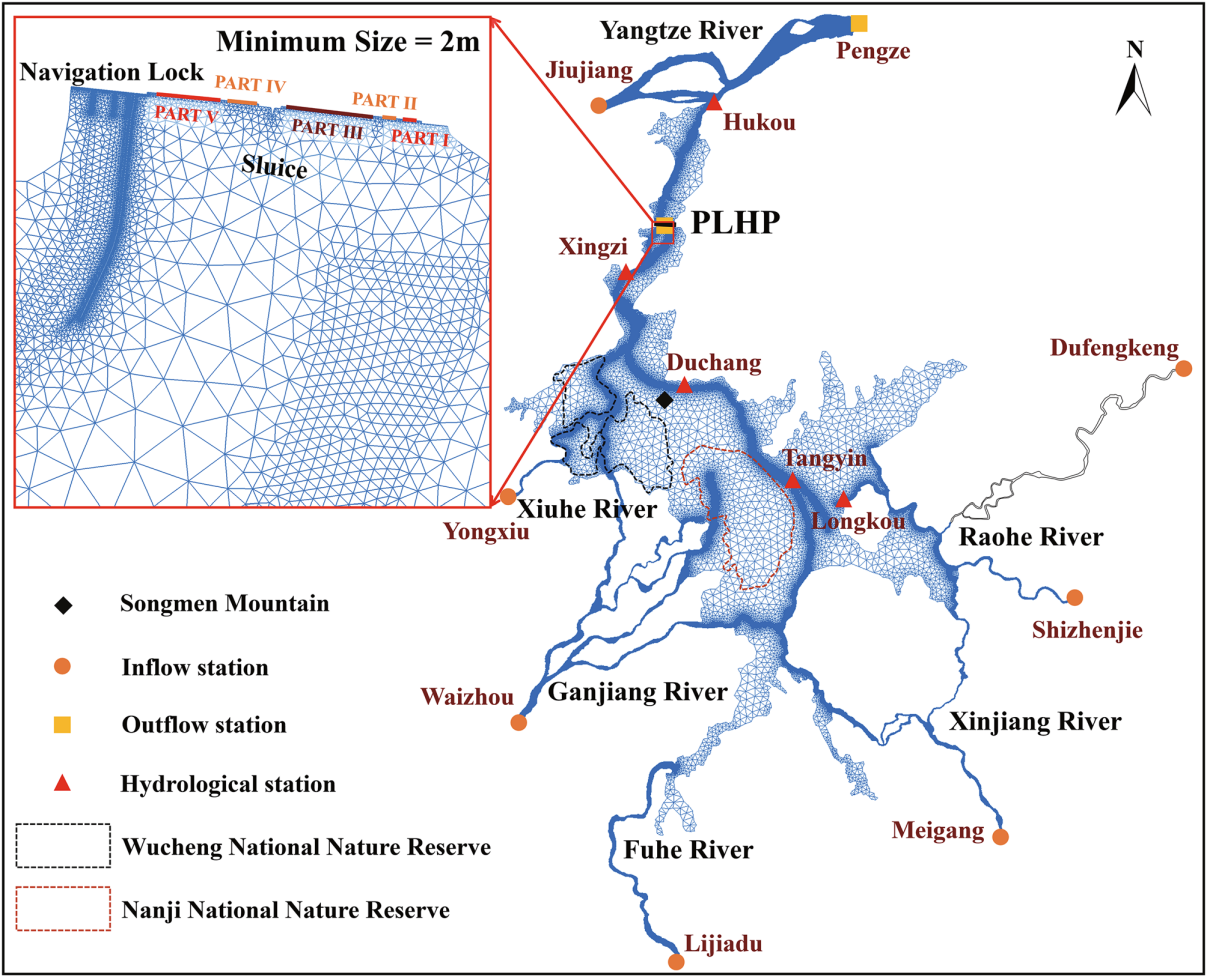
Poyang Lake with locations of hydrological stations, nature reserves, the PLHP, the computation domain and the closeup of the computational mesh near the PLHP. The figure was generated by BlueKenue 3.3.4 (https://nrc.canada.ca/en/research-development/products-services/software-applications/blue-kenuetm-software-tool-hydraulic-modellers).

Poyang Lake is located in the north of Jiangxi Province, on the southern bank of the middle to lower reaches of the Yangtze River. Bounded by Songmen Mountain, Poyang Lake is divided into two parts. The northern part is the waterway that connects Poyang Lake and the Yangtze River, while the southern part is the main region of the lake. As shown in Fig. 1, the northern waterway connects to the Yangtze River at Hukou. The southern part of Poyang Lake receives water mainly from five tributaries, including the Xiuhe River, Ganjiang River, Fuhe River, Xinjiang River, Raohe River (known as the Five Rivers). The Ganjiang River is the largest river among the Five Rivers and contributes about 55% of the total flow rate into the lake^22^.

Corresponding to the large water level fluctuation, The inundation extent of Poyang Lake varies greatly with seasons^17^, from less than 1000 km^2^ to more than 3000 km^2^^23^. As a result, Poyang Lake appears as a network of rivers in the dry season and a single interconnected water body in the rainy season. Such seasonal variation produces a large number of temporary wetlands with various water depths, which contributes to the abundance of different species of plants, fish and birds. According to statistics, approximately 400,000 migratory birds spend winter here each year, including nearly 98% of the world’s Siberian Crane, 60% of the Wild Geese, 50% of the White-naped Crane and other endangered or vulnerable species^24,25^. Therefore, Wucheng National Nature Reserve and Nanji National Nature Reserve have been established to protect the biodiversity and the habitat of these migratory birds. (Fig. 1).

### The PLHP and its regulatory scheme

There has been a persistent shrinkage of the water volume in Poyang Lake since the operation of the Three Gorges Dam (TGD) ^26,27^. The PLHP was proposed to alleviate the negative ecological and social effects that might be caused in case of the drought^28^. According to the latest official announcement of the design, the proposed PLHP will be established at the narrowest location of the waterway that connects Poyang Lake and the Yangtze River, with a width of 2.8 km. This project is mainly composed of a navigation lock on the left side and a series of sluices on the right side. The sluices have 64 holes and are divided into five parts (Fig. 1).

The latest published regulatory scheme of the PLHP follows a principle of “zero control in the flood season and limited regulation in other times”. Those sluices will remain completely open from April to August in the flood season, while during the rest of the year the degree of the sluices’ opening will change to maintain a suitable water level in the lake. The detailed scheme is shown in Table 1.

**Table 1:** Regulatory scheme of the PLHP.

Date	Regulation goals
April 1st–August 31st	All sluices are open so that water exchanges freely between the Yangtze River and Poyang Lake
September 1st–September 15th	Adjust the water level upstream of the PLHP to 14.20 m
September 16th–January 10th	Reduce the water level upstream of the PLHP to 7.10 m
January 11th–March 31st	Increase the water level upstream of the PLHP to 10.23 m

The elevations in the table and hereinafter refer to the Yellow Sea datum; In the regulatory scheme, the ecological flow rate is 803 m^3^/s.

## Materials and methods

### Governing equations

Poyang Lake is wide and shallow, so vertical stratification can be ignored. In this research, the TELEMAC-2D software has been used to establish the two-dimensional hydrodynamic model of Poyang Lake. TELEMAC is a powerful integrated modelling tool for simulating river systems, shallow lakes and estuaries^29–32^. It solves depth-averaged free surface flow equations first derived by Barré de Saint–Venant, which include continuity and momentum equations.

The continuity equation in the non-conservative form is as follows.

1 $$\frac{\partial h}{{\partial t}} + {\varvec{u}} \cdot \nabla (h) + hdiv({\varvec{u}}) = S_{h}$$

The momentum equations in the non-conservative form are as follows:

2 $$\frac{\partial u}{{\partial t}} + {\varvec{u}} \cdot \nabla (u) = - g\frac{\partial Z}{{\partial x}} + S_{x}^{{}} + \frac{1}{h}div(h\nu_{t} \nabla u)$$

3 $$\frac{\partial v}{{\partial t}} + {\varvec{u}} \cdot \nabla (v) = - g\frac{\partial Z}{{\partial y}} + S_{y}^{{}} + \frac{1}{h}div(h\nu_{t} \nabla v)$$

where *h* is water depth, *t* is time, *u* and *v* are depth-averaged velocity components along the two Cartesian coordinates *x* and *y*, respectively, ***u*** = (*u*,*v*) is the vector of the velocity, *g* is gravitational acceleration, *v*_*t*_ is eddy viscosity coefficient, *Z* is free surface elevation, *S*_*h*_ is source or sink term in the continuity equation, *S*_*x*_ and *S*_*y*_ are bed friction contributions in the *x*-direction and *y*-direction, respectively. In the present study, *S*_*h*_ = 0, and Manning coefficient is chosen to describe bed friction, as a result, *S*_x_ and *S*_y_ can be expressed as follows:

4 $$S_{{\text{x}}} = - \frac{u}{\cos (\alpha )}\frac{{gm^{2} }}{{h^{{{4 \mathord{\left/ {\vphantom {4 3}} \right. \kern-\nulldelimiterspace} 3}}} }}\sqrt {u^{2} + v^{2} }$$

5 $$S_{{\text{y}}} = - \frac{v}{\cos (\alpha )}\frac{{gm^{2} }}{{h^{{{4 \mathord{\left/ {\vphantom {4 3}} \right. \kern-\nulldelimiterspace} 3}}} }}\sqrt {u^{2} + v^{2} }$$

where *α* is slope angle, *m* is Manning coefficient.

### Description of Poyang Lake models

Two hydrodynamic models are established to investigate the influence of the PLHP on the hydrodynamic conditions in the lake region. The first one (M1) is the Poyang Lake hydrodynamic model without the PLHP. In this model, the mesh size ranges from 50 m in the tributary rivers and main channels to 1500 m in the middle of the Lake. A total of 199,103 nodes and 377,157 triangular elements are used to discretize the studied domain, which has an area of approximately 3496.94 km^2^. The model’s inflow boundary conditions are specified according to the daily flow rate data in the Five Rivers and the Yangtze River, which have been recorded by seven hydrological stations. Those hydrological stations include Jiujiang for the Yangtze River, Yongxiu for the Xiuhe River, Waizhou for the Ganjiang River, Lijiadu for the Fuhe River, Meigang for the Xinjiang River, Shizhenjie and Dufengkeng for the Raohe River. The outflow boundary condition is specified according to the daily water level records at Pengze Station.

The second model (M2) is the Poyang Lake hydrodynamic model with the presence of the PLHP. Compared with M1, the computational mesh needs to be refined near the PLHP site to resolve the structural details according to the design of the PLHP. A total of 179,518 nodes and 338,984 triangular elements are used to discretize the computational domain, which has an area of approximately 3159.04 km^2^. M2 has the same inflow boundaries on the Five Rivers as in the case of M1, while its outflow boundary is set up at the location of the PLHP. The computational mesh and boundaries can be seen in Fig. 1, with the enlarged inset illustrating the mesh refinement condition near the PLHP in M2.

### Validation of the model

**Figure 2:**
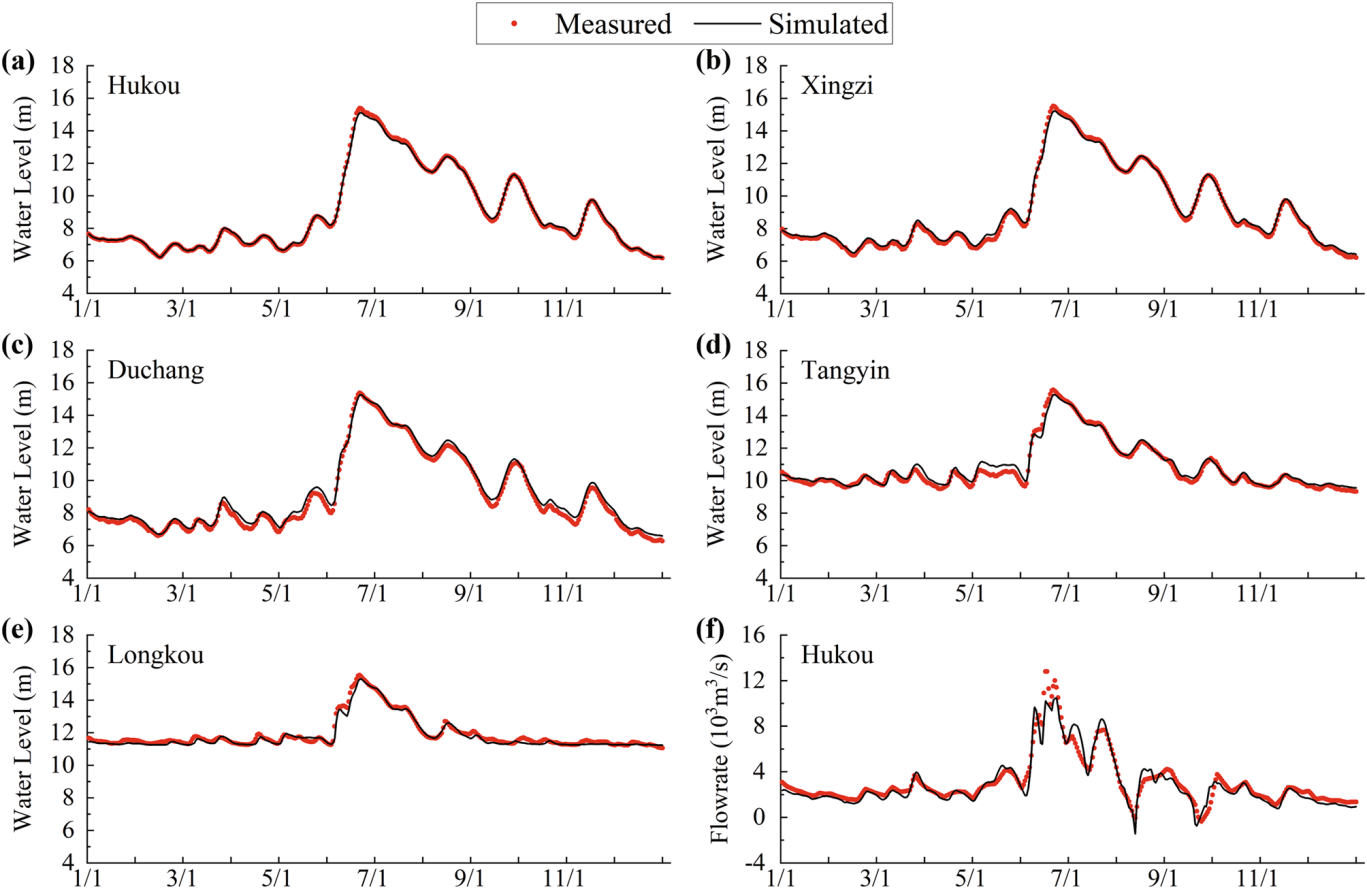
Comparisons between measured and simulated daily water levels and flowrates in 2011.

The topography of Poyang Lake in 2010 were obtained to establish the present models, and the 2011 data were used for validation. Prior to the formal simulation, constant flow rates and water levels at the beginning of the year were specified for 40 days in the model’s warm-up period. For model validation, the computed values were compared with the measured water level at the five hydrological stations (Hukou, Xingzi, Duchang, Tangyin and Longkou as shown in Fig. 1) and the measured flowrate at Hukou Station. Figures 2a ~ e show the comparison of computed and measured water levels, while Fig. 2f shows the comparison of flowrates.

The mean absolute error, root mean square error (RMSE) and Nash–Sutcliffe coefficient were calculated and tabulated in Table 2 to quantitatively analyze the discrepancy. For the water levels at the five hydrological stations, the mean absolute errors range from 0.068 m to 0.241 m, the RMSE range from 0.088 m to 0.273 m, and the Nash–Sutcliffe coefficients range from 0.972 to 0.999. The consensus is that the goodness of fit can be considered as very good when the Nash–Sutcliffe coefficient is between 0.75 and 1^33^. Hence, it can be said that the present model can generally reproduce the hydrodynamic process of Poyang Lake. After validation, the Manning coefficient was determined to be 0.022 and the eddy viscosity coefficient was taken to be 1.0 m^2^/s.

**Table 2:** Error analysis for model calibration.

	Stations	Mean absolute error	RMSE	Nash–Sutcliffe coefficient
Water level	Hukou	0.068 (m)	0.088 (m)	0.999
	Xingzi	0.139 (m)	0.162 (m)	0.996
	Duchang	0.241 (m)	0.273 (m)	0.986
	Tangyin	0.180 (m)	0.235 (m)	0.975
	Longkou	0.118 (m)	0.161 (m)	0.972
Flowrate	Hukou	439.006 (m^3^/s)	614.231 (m^3^/s)	0.921

The difference between the simulated results and the measured results can be caused for many reasons. For example, illegal sand dredging activities can cause dramatic changes in the topography and bathymetry of the region. Furthermore, some interpolations are needed as the field survey was conducted only in discrete locations and cross-sections, which can also result in deviations from reality.

### Habitat model for *Vallisneria natans*

*Vallisneria natans* is a stoloniferous submersed macrophyte. Its growing season spans from March to October. *Vallisneria natans* exists widely in natural aquatic areas. It can reproduce through both sexual and asexual reproduction by seeds or rhizomes^34^, and serve as food sources and habitats for aquatic animals^35^. In Poyang Lake, it mainly distributes in the lakeside zone with water depth less than 4 m^16^.

**Figure 3:**
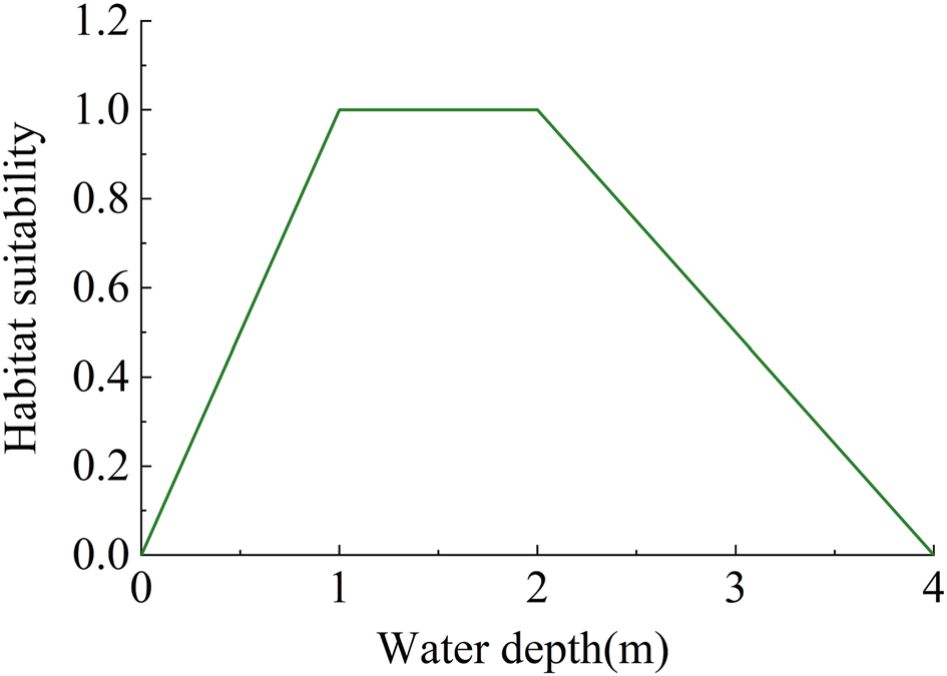
Habitat suitability for *Vallisneria natans* with water depth^16^.

The growth of *Vallisneria natans* is directly related to the water depth ^16,36,37^, and the correlation between its density and the water depth can successfully explain the locations of 75% of the feeding grounds of Siberian Crane. According to the study of Chen et al.^16^, water depths between 1 and 2 m are the most favorable condition for the growth of *Vallisneria natans*. Other water depths will lead to either too much or too little sunlight and thus inhibition of growth. On this basis, they established a correlation between the level of suitability for the growth of *Vallisneria natans* and the water depth. Indicators between 0 and 1 were used to describe the preference degree, with larger values representing higher degrees of suitability for the survival of the species. As shown in Fig. 3, the suitability of growth for *Vallisneria natans* increases first and then decreases with the water depth. The suitability reaches the maximum value when the water depth is between 1 and 2 m. When the water depth is greater than 4 m, the suitability decreases to 0, which means that *Vallisneria natans* stops growing in such deep waters. This habitat model has been used and verified in the research of Zhu et al.^15^.

In order to quantify the habitat suitability of *Vallisneria natans*, the concept of habitat area is introduced by Chen et al.^16^. The computational formula proposed by them is shown below:

6 $$WUA = \sum\limits_{{{\text{i}} = 1}}^{{\text{n}}} {SI_{i} A_{i} }$$

where *WUA* is the weighted available habitat area (km^2^), *A*_*i*_ and *SI*_*i*_ are the area and habitat suitability of the *i*_th_ area in the considered domain.

### Scenario simulation scheme

After the validation of the model, the M1 and M2 are used to predict the possible impact of the PLHP on the water depth and thus the habitat suitability of *Vallisneria natans* in Poyang Lake. The water level recorded at Xingzi station in September and October from 2003 to 2019 are analyzed for the scenario design. The average water levels during these periods are calculated and ranked, and 2006, 2018 and 2010 are chosen as the typical years with a low water level, medium water level and high water level, respectively.

The water levels at the PLHP in M2 are constructed by M1 and the designed water levels specified in the regulatory scheme. According to the regulatory scheme, the water levels in M2 should be gradually adjusted to 14.2 m before the 15th of September. It is important that the outflow should satisfy the ecological requirements in the downstream area, so the minimum outflow rate is 803 m^3^/s.

**Figure 4:**
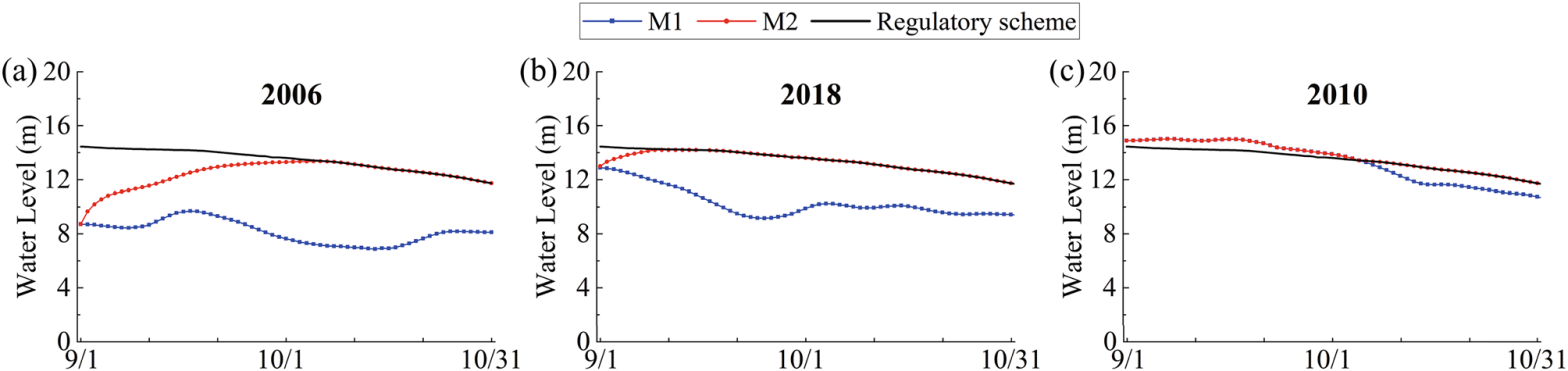
The water level variations upstream of the PLHP during September and October in typical years.

Because the PLHP is not supposed to regulate the flow from April to August and the main growth period of *Vallisneria natans* is before November, we hereby only focus on the changes in the water depth and habitat suitability from the 1st of September to the 31st of October. Based on the water level variations specified in the regulatory scheme (as shown by the black lines in Fig. 4), the water levels upstream of the PLHP in three typical years are adjusted (as shown by the red lines in Fig. 4). At the beginning of the regulation period in the low-water-level year (2006), the PLHP should prioritize the ecological outflow while raising the water level. Consequently, the water level cannot reach the target value as specified in the regulatory scheme until the 6th of October. At the beginning of the regulation period in the medium-water-level year (2018), the water level rises to 14.2 m on the 9th of September. Then, the opening degree of the sluices is adjusted to achieve a constant water level by ensuring the outflow rate and inflow rate to be the same. After the 15th of September, the water level is adjusted in accordance with the regulatory scheme. In the high-water-level year (2010), the water level at the beginning of the regulation period is higher than 14.2 m. Therefore, all sluices are fully open and allow the free water exchange, leading the water level to fall to satisfy the regulatory scheme. As a consequence, M2 has the same water level as M1 from the 1st of September to the 5th of October.

## Results and discussions

### Influence of the PLHP on water depth distribution

**Figure 5:**
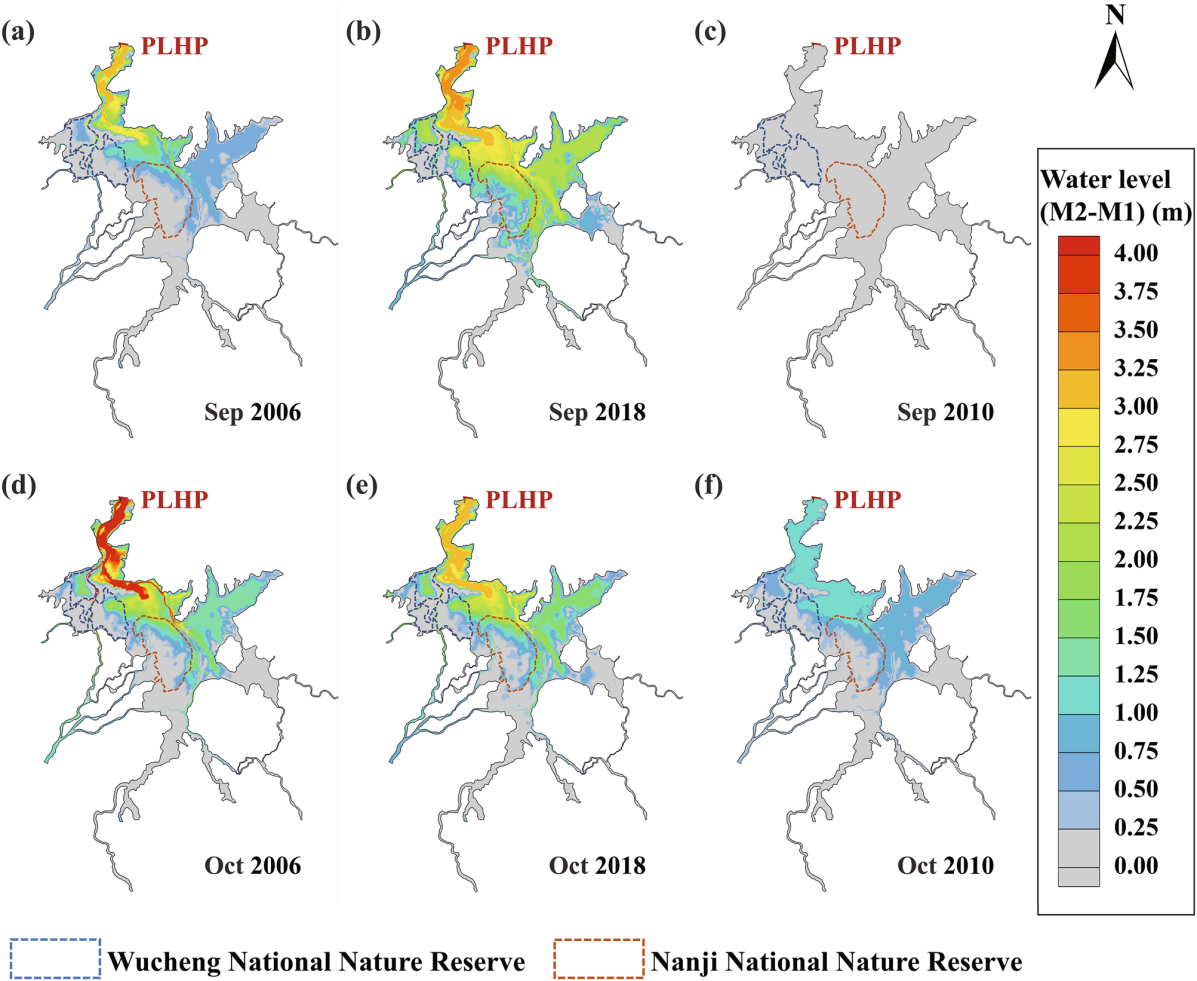
Water depth variation during September and October in the typical years. The figure was generated by Tecplot2020 (https://www.tecplot.com/).

The hydrodynamic process of Poyang Lake with and without the PLHP is simulated by M1 and M2, respectively. By comparing and analyzing the simulation results, we obtain the changes of the water depth, i.e., the water depth in M2 minus the water depth in M1, in Poyang Lake. Figure 5 shows the mean monthly water depth differences during September and October in three typical years. The water depth in Poyang Lake has increased obviously in most cases after the operation of the project. Combining the water level processes in Fig. 4 and the water depth differences in Fig. 5, the area of the changes in the water depth is seen to be mainly controlled by the Higher Water Levels (HWL) between M1 and M2. While the magnitude of the changes is mainly controlled by the Differences in Water Levels (DWL) between M1 and M2.

As shown in the water level variations processes in Fig. 4a, in the low-water-level year (2006), where the natural inflow is relatively small, the water level in M2 is much higher than that in M1 and reaches the peak value around 10th of October. The DWL also reaches maximum in this time and then gradually decreases. As a consequence, the water depth increases the most in October 2006. As shown in Fig. 5d, in most areas of Poyang Lake, the Xiuhe River and the Ganjiang River, the water depth is increased by more than 1 m. Especially in the main channel from the PLHP to Tangyin and Wucheng, the increase can exceed 4 m, as shown in the red parts in Fig. 5d. While over the surrounding flooded land, the increase ranges from 1 to 3 m. The increase in the water depth during September was essentially the same as that in October, but the area and magnitude of the changes are slightly reduced. The maximum increase is about 3.1 m, which is mainly concentrated in the main channel on the north of Songmen Mountain.

In the medium-water-level year (2018), the water level in M1 is constantly lower than that in M2 during September and October, which can be seen in Fig. 4b. Both the HWL and DWL first increase and then decrease, reaching their maximum values in early September and late September, respectively. Furthermore, the mean monthly HWL and DWL in September are slightly greater than those in October. As a consequence, the area and magnitude of the changes in Fig. 5b are larger than those in Fig. 5e. In September 2018, almost the entire main lake region becomes influenced by the PLHP and the increase in water depth ranges from 0 to 3.4 m. While in October 2018, the increase in water depth ranges from 0 to 3.2 m, and the significant increase is mainly concentrated in the main channel on the north of Songmen Mountain.

In the high-water-level year (2010), as shown in Fig. 4c, the HWL rises and the DWL decreases as compared to those in other years. During the first 35 days, the hydrodynamic conditions in M2 are exactly the same as that in M1, so the PLHP has no effect on the water depth in the lake region in September (Fig. 5c). However, after the 6th of October, the water level in M2 begins to be higher than that in M1 under the regulation of the PLHP. Although the DWL is small during this time, the HWL is relatively high and thus results in large areas of water depth increase, as shown in Fig. 5f. The maximum increase in water depth is approximately 1.1 m.

In these two months, the northeastern parts of the two national nature reserves are observably influenced., The area of the changes is greatest in September 2018, there has been a marked increase in water depth in most areas of the reserves, with the maximum increase reaching about 2.8 m. While in other periods during September and October, with the exception of September 2010, the water depth is increased significantly in about 1/3 ~ 1/2 area of the reserves, with the maximum increase reaching about 2.6 m. In the meantime, the water depth in the southwestern part of these two reserves remains essentially the same as before the operation of the PLHP, with an increase of less than 0.25 m as shown in the grey parts in Fig. 5.

### Influence of the PLHP on habitat suitability of Vallisneria natans

**Figure 6:**
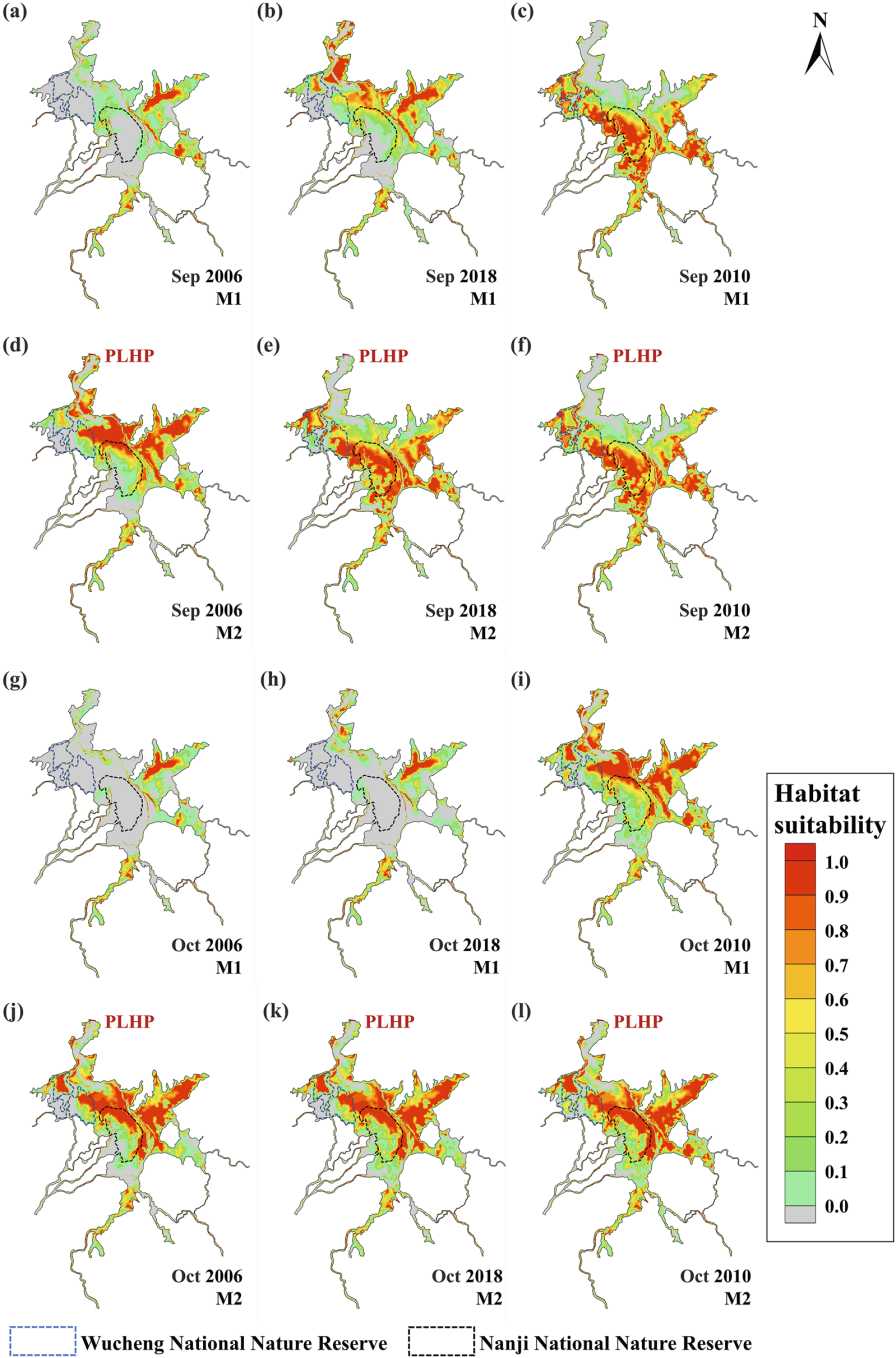
Distributions of the habitat suitability of *Vallisneria natans* in typical years without (the first and third rows) and with (the second and fourth rows) the PLHP. The figure was generated by Tecplot2020 (https://www.tecplot.com/).

According to the relationship between the habitat suitability of *Vallisneria natans* and the water depth as mentioned in Fig. 3, the water depth results can be translated into the habitat suitability of *Vallisneria natans*, as shown in Fig. 6. The first and third rows are the distributions of the habitat suitability during September and October, respectively, in the three typical years before the operation of the PLHP, while the second and fourth rows are distributions of the habitat suitability after the operation of the PLHP. The grey parts in Fig. 6 indicate that the habitat suitability is 0, implying that the area is dry or the water depth is greater than 4 m.

As shown in Fig. 6, the suitable area for the growth of *Vallisneria natans* is mainly concentrated over the flooded land. As the water depth in the main channel is generally more than 4 m, so the habitat suitability is usually 0 there.

Before the operation of the PLHP (M1), the water level in 2010 is higher than that those in 2006 and 2018. Therefore, there are more areas covered by water in 2010, and the habitat suitability in Fig. 6c is greater than those in Fig. 6a,b. Similarly, the habitat suitability in Fig. 6i is greater than those in Fig. 6g,h. Because the lake bed is lower in northeastern part than that in the southwestern part, the water depth generally decreases from northeast to southwest. During September and October in 2006 and 2018, large areas of bed in the northeastern part are covered by water, and the water depth is less than 4 m, which is suitable for the growth of *Vallisneria natans*. While large areas in the southwestern part of the lake are dry, and thus the habitat suitability is 0, as shown in the grey parts in the southwestern part of the main lake region in Fig. 6a,b,g,h. However, because the water level is relatively high during September and October in 2010, there are almost no dry areas in the lake region. As a result, with the exception of the main channel, where the water depth is greater than 4 m, most of the areas in the lake region are suitable for the growth of *Vallisneria natans*. The most suitable areas for *Vallisneria natans* vary between these two months, as the water depth in September 2010 is greater than that in October 2010. As shown in Fig. 6c, the red parts are mainly concentrated in the southwestern part of the lake, because there are large areas of flooded region with water depths ranging from 1 to 2 m, which is ideal for the growth of *Vallisneria natans*. On the contrary, the water depth in the northeastern flood land is usually between 2-4 m. In Fig. 6i, however, the red parts are mainly concentrated in the northeastern part of the lake, because the water depth there is usually between 1 and 2 m, and the water depth in the southwestern flood land is now usually less than 1 m.

After the operation of the PLHP (M2), with the rise of the water level in the lake region, the suitable area for the growth of *Vallisneria natans* is increased greatly, and the variation is proportional to the DWL during the same period. The increase is most obvious in the low-water-level year (2006), followed by the medium water level year (2018), and becomes insignificant in the high-water-level year (2010). Such a trend is consistent with the previously mentioned variation in the water depth.

Since the DWL is relatively small during September and October in 2010, the habitat suitability in this period is changed little between M1 and M2 (Fig. 6). The distribution of the habitat suitability is completely the same between Fig. 6c,f, while the difference in the distributions of habitat suitability between Fig. 6i,l is subtle. As the water level in M1 is relatively low during September and October in 2006 and 2018, the suitable area for the growth of *Vallisneria natans* in M2 is expanded from northeast to southwest under the influence of the PLHP. In addition, the habitat suitability is increased greatly in Wucheng National Nature Reserve and Nanji National Nature Reserve. Before the operation of the PLHP, there are large areas of dry land in the two reserves, and thus the habitat suitability in these dry areas is 0. After the operation of the PLHP, according the previous research, more than 1/3 of area in the two reserves sees apparent increases in water depth. The water depth is increased to 1 ~ 2 m and thus the habitat suitability is increased to 1.0 in the northeastern part of the reserves. In the southwestern part of the reserves, although the increase of water depth is not significant, most of the lake bed has changed its status from being dry to being wet and the habitat suitability is correspondingly increased from 0 to 0.1 ~ 0.2, as shown in Figs. 6d,e,j,k. This means that the two nature reserves will be more suitable for the growth of *Vallisneria natans* after the operation of the PLHP, and there it will be easier for Siberian Crane to find food here.

### Changes in habitat area of Vallisneria natans

**Figure 7:**
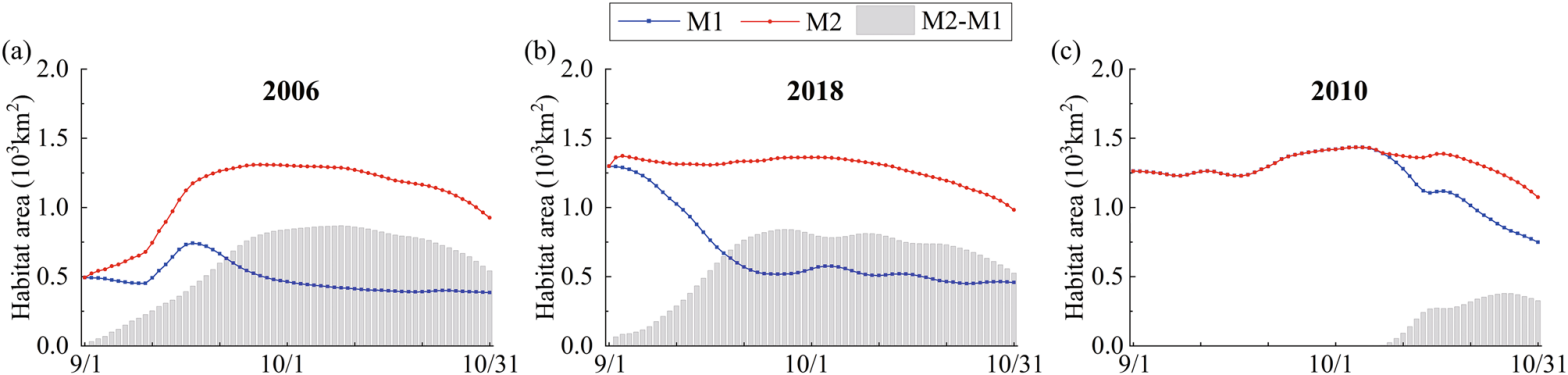
Variations of habitat areas of *Vallisneria natans* in M1 (without the PLHP) and M2 (with the PLHP) and the resulting differences (grey columns).

On the basis of formula (6), we calculate the habitat area from 1st of September to 31st of October in three typical years, as shown in Fig. 7 and Table 3. In 2006, the habitat area is increased greatly, and the impact of the PLHP on habitat area is most evident in October. Compared with M1, the mean monthly habitat area in M2 is increased by 190.92%. Especially around the 10th of October, the increase can reach about 867.79 km^2^, accounting for about 1/4 of the total area of the lake. Compared with 2006, the increase of habitat area in 2018 is smaller. The mean monthly habitat area in M2 is increased by 57.07% in September and by 145.27% in October. The largest change occurs in late September, with an increase of about 841.43 km^2^, which is basically the same as in 2006. While in 2010, compared with M1, the habitat area in M2 is completely the same in September and the average increase in October is only 18.07%, indicating that when the water level is relatively high the operation of the PLHP will make little change to the habitat area.

**Table 3:** Mean monthly habitat areas of *Vallisneria natans* and changes between M1 and M2.

Typical years	September in M1 (km^2^)	September in M2 (km^2^)	Δ (km^2^)	Δ/M1 (%)	October in M1 (km^2^)	October in M2 (km^2^)	Δ (km^2^)	Δ/M1 (%)
2006	559.28	975.84	416.56	74.48	409.04	1189.96	780.93	190.92
2018	849.87	1334.91	485.04	57.07	502.62	1232.79	730.17	145.27
2010	1289.74	1289.74	0	0	1128.37	1332.23	203.86	18.07

After the operation of the PLHP, the habitat area can reach more than 1000 km^2^ during the most time of September and October in three typical years, accounting for about 1/3 of the total area of the lake region. In other words, the latest official regulatory scheme of the PLHP is beneficial for the growth of *Vallisneria natans* in September and October. It means that, whether it is a low-water-level year, medium-water-level year or high-water-level year, before Siberian Crane fly to Poyang Lake for winter, *Vallisneria natans* will occupy large areas of the flooded land under the regulation of the PLHP. It will ensure that Siberian Crane can consume abundant tubers of *Vallisneria natans* as food in winter, allowing them to better survive and reproduce in the wetlands of Poyang Lake.

In this research, the habitat suitability model of *Vallisneria natans* in Poyang Lake is established based on the previous research of Chen et al.^16^. This model only considers the effect of water depth, which mainly influences the growth of aquatic plant by changing the degree of light attenuation^34^. In fact, temperature and flow velocity can also influence the growth of *Vallisneria natans* according the relevant studies. However, temperature only plays a decisive role in the germination period^38^ and is rarely considered as an influencing factor during the growth period of *Vallisneria natans*. While high flow velocity may adversely affect the growth of *Vallisneria natans* during the seedling period, adult *Vallisneria* plants have an extensive root–rhizome system and long ribbon-like leaves to prevent them from being torn apart in rapid water^39^. *Vallisneria natans* often begin to sprout in March, reaching the tillering stage in June or July in Poyang Lake region. As a consequence, the temperature and flow velocity will have little effect on the growth of *Vallisneria natans* during the study period in this research (September and October). Therefore, the water depth can be regarded as the main factor affecting the suitability of *Vallisneria natans* during the mature period.

There have been several scholars who have studied the effect of water depth on the growth of *Vallisneria natans* in other areas. Xiao et al.^40^ reported that *Vallisneria natans* grow rapidly with depths of 110–160 cm, while the growth is severely retarded with a depth of 250 cm. Cao et al.^34^ carried out experiments in turbid water and reported that the water depth of about 130 cm is most suitable for the growth of *Vallisneria natans*, while higher water depth will be less favorable for the growth. The inconsistency between these findings and the habitat suitability curve proposed by Chen et al.^16^ may be explained by the climate differences in different regions, as well as the different degrees of turbidity in water. According to the above analysis, the habitat suitability model of *Vallisneria natans* in this paper is established based on the habitat suitability curve proposed by Chen et al. (2020) as it represents to the growth characteristics of *Vallisneria natans* in Poyang Lake region.

According to the above analysis, the latest regulatory scheme of the PLHP will effectively increase the water level in the lake region and expand the habitat area of *Vallisneria natans*, especially in low-water-level years. This finding is different from some of the previous research. Zhu et al.^15^ used the average water depth during the growing period of *Vallisneria natans* (from March to October) to reflect the availability of this food resource for Siberian Crane. They found that the PLHP had few influences on *Vallisneria natans*. This was because the PLHP remain completely open from April to August and it takes effect only in March, September and October during the growing period of *Vallisneria natans*. Therefore, the average water depth during March to October differs little whether with or without the PLHP, which would certainly underestimate the impact of the PLHP. The present study focuses on September and October, and uses the mean monthly water depth to reflect the habitat suitability of *Vallisneria natans*. In this period, the natural water level is relatively low in M1, and the operation of the PLHP increases the water level and inundates large areas of otherwise dry land. As a result, the habitat areas of *Vallisneria natans* observe an increase.

## Conclusions

As the main food source for Siberian Crane, an indicator species of the wintering migratory birds in Poyang Lake, *Vallisneria natans* habitat can directly determine whether Siberian Crane can get enough food to survive in winter. However, the influence of the PLHP on the habitat of *Vallisneria natans* is still unclear. Here, we used TELEMAC-2D software to establish a two-dimension hydrodynamic model of Poyang Lake without the PLHP (M1) and with the PLHP (M2). Then, we simulated the hydrodynamic process during September and October in three typical years (2006, 2018 and 2010). The simulation results showed that, most likely after the operation of the PLHP, more than half of the area showed significant increase in the water depth by more than 0.25 m. Especially in the main channel north of Songmen Mountain, the maximum increase can exceed 4 m during the low-water-level year (2006). For Wucheng National Nature Reserve and Nanji National Nature Reserve, in most cases, more than 1/3 of the area showed a large increase in the water depth. The maximum can exceed 2.8 m, which mainly concentrated in the northeastern part of the reserves. With the increase in the water depth, the suitable area for the growth of *Vallisneria natans* in M2 can increase significantly and expand from northeast to southwest in Poyang Lake compared with the situation in M1. The mean monthly habitat area can increase by up to 190.92% in the low-water-level year, 145.27% in the medium-water-level year, yet only 18.07% in the high-water-level year. The habitat area can reach more than 1000 km^2^ in most times. This means that after the operation of the PLHP, *Vallisneria natans* will gain large areas in the lake region suitable for its growth.

The growth of *Vallisneria natans* is long-term and continuous. Therefore, it is necessary to consider the complete growth cycle of *Vallisneria natans* to understand the complete effects of the PLHP. More importantly, *Vallisneria natans* is only one of the factors that affect the survival of Siberian Crane. Consequently, the ecological impact of the PLHP on *Vallisneria natans* and Siberian Crane requires further research.

## Consent to Publish

The authors declare that they consent to publish this manuscript.

## Data Availability

Available upon request.
